# 

**Published:** 1891-02

**Authors:** 


### LITERARY.

TEXT BOOK OF HYGIENE.—PRINCIPLES and PRACTICE of PREVENTIVE MEDICINE.—By George H. Rohé, M. D. Second Edition, 1890. F. A. Davis, Publisher, Philadelphia. Price $2.50.

This is a volume of 421 pp. upon the best means of securing and preserving good health.

It treats in a most comprehensive manner, of all the well known elements requisite for human sustenance, giving the causes and effects of their poisonous degeneration, as well as the simple and effectual means of their purification, as well in private homes as in places of public resort.

It also treats of the hygiene of cities—those populous centres necessitating the most ample provisions against the ingress of infections and contagions so destructive of human life, explaining the most approved Sanitary methods of constructing and operating the various sub and superstructures so essential to even a partial security against the inroads of disease.

Considered as a whole, this work covers the entire ground of hygienic science, applicable to individuals and communities, and ought to be in the hands of all persons whose calling lies more or less in that important field.

We are indebted to the several authors and publishers for the following works :

REVUE INTERNATIONALE DE BIBLIOGRAPHIE MÉDICALE, PHARMACEUTIQUE ET VÉTÉRINAIRE, dirigée par Le Docteur JULES ROUVIER, Professeur, etc., Beyrouth, Siria, Vol. 11, No. 4 , Oct., 1890.

THE PRESCRIPTION (Vol. 1., No. 1), a monthly Journal devoted to Practical Therapeutics, issued by the Danbury Medical Printing Co., Danbury, Conn. ; price $1 per annum.

This thoroughly practical and professional undertaking, cannot fail to be appreciated and sustained by medical practitioners.

The Prospectus and Sample Pages of Funk & Wagnall's STANDARD DICTIONARY OF THE ENGLISH LANGUAGE, 18 and 20 Astor Place, New York City.

A literary and scholastic undertaking of great magnitude and importance to the English speaking race. The work, now fairly under way, promises to be a most complete cyclopedia of our language, treating of words not only etymologically and philologically, but by example in respect to their use by the more eminent writers of this and other times, and containing also the completest dictionary of quotations from the most renowned authors which has yet been produced. The value of such a work can hardly be estimated. It is not a little singular that the acknowledged best two modern Dictionaries of our language have been compiled and published by Americans, and now we are to have a third which in point of thoroughness will at least equal, if not excel, the Websters and Worcesters so familiar to our libraries. We shall look for its advent with undiminished interest.

FIRST HOLIDAY EDITION OF THE FAIRHAVEN HERALD, WASHINGTON.

When we consider that the handsomely printed and finely and profusely illustrated mammoth newspaper before us, of 24 well filled pages (14 x 21 in.), comes from a town in the newly organized State of Washington, whose beginning was only fifteen months ago, it deserves to rank among the "curiosities of literature." The town and people capable of such master strides are fully equal o achieving and maintaining a prominent place among the rival centres of the great and growing west.

GOLDTHWAITE'S GEOGRAPHICAL EXCHANGE, Vol. 1, No. 1, 117 Nassau street, New York City.

This is a new Illustrated monthly of 85 pp., devoted to geographical explorations, discoveries and information ; the first of this exclusive kind ever undertaken ; price $2.00 per annum. It merits a liberal patronage.

EFFECT OF HIGH EXPLOSIVES—DYNAMITE AND NITRO-GLYCERINE, on the human system ; by Thomas Darlington, M. D., New York City.

This is an eight page pamphlet, highly interesting as treating upon a subject quite original.

REMOVAL OF TONSILLAR HYPERTROPHY, BY ELECTRO-CAUTERY DISSECTION, By Edwin Pynchon, M. D., Chicago; 12 pp.

BULLETIN OF THE AMERICAN ACADEMY OF MEDICINE, January, 1891.

This pamphlet is particularly interesting as containing a comparative table showing the variations of the academic course of forty American Colleges, especially in respect to dead and living foreign languages.

ABNORMAL INTRA-THORACIC AIR-PRESSURES AND THEIR TREATMENT. An address delivered at the Seventh Annual Meeting of the American Climatological Association, 1890, by Professor Charles Denison, A. M., M. D. Colorado, Prest. An illustrated pamphlet of 41 pp., replete with recitals of individual cases.

INSPIRATIONS OF THE SCHOOL-TEACHER. This is an unpretentious little book by a practical teacher, of value to all classes of readers. W. W. Knowles & Co., Chicago.

CALENDARS, 1891.—There may be a more uniquely elegant Calendar somewhere published, than that which found its way to our sanctum, by the favor of its enterprising publishers, Mess. Lea & Shepard, of Boston, but certain it is that none such has come under our observation. The illustrated catalogue of infant periodicals and toy books which accompanied it, is a fit companion piece in the exquisite nature of its illustrations, typography, and arrangement. It is hardly possible to conceive any thing more artistically perfect in its line.

GALAXY OF MUSIC, for January. E. Trifet, Boston. This publication contains 14 select pieces of music, vocal and instrumental, published monthly, $1.00 per annum. At this rate the yearly subscriber would get some 175 pieces of music arranged in two or more parts, at less than three-quarters of a cent per copy.

THE OPEN COURT PUBLISHING Co., of Chicago, are about to publish in two handsome volumes, a new authorized translation of Gustav Freytag's well known novel, "The Lost Manuscript." This is regarded by critics as the writer's best work.

"COLLEGE AND SCHOOL" monthly has been sold by Mr. F. G. Barry, to Louis Lombard, of Utica, N. Y. The next number will appear February 15, entitled *The Louis Lombard*, with a guaranteed circulation of 5,000 copies and a list of able contributors, comprising many well known writers.

PROF. KOCH'S METHOD TO CURE TUBERCULOSIS, popularly treated by Dr. Max Birnbaum. Translated from the German by Dr. Fer. Brendecke, with an appendix, etc., Milwaukee, Wis. H. E. Haferkorn, publisher, 1891, 27 pp., illustrated. Price, 75 cents, cloth $1.00.

Since the year 1882, when Prof. Koch published to the world his discovery that some of the worst forms of disease are attributable to micro-organisms, persistent efforts have been made to provide some means of eradicating the evil, and it was fitting that the eminent discoverer, in the first instance, should also be the one to supplement his work by this additional discovery. The treatise before us, is the completest that has appeared in this country, on the symptoms, ravages and effect of tuberculosis, and the treatment by Prof. Koch's method, for the expulsion of microbes, with suggestions for the prevention of the evil, through proper sanitary measures. Physicians will find it of great value to them in their practice.

We are also indebted to THE ARLINGTON CHEMICAL Co., Yonkers, N. Y., for a large size portrait of Prof. Koch, and many interesting particulars of his life and labors, especially in respect to those discoveries which have made his name a household word throughout the civilized world.

VICK'S FLORAL GUIDE FOR 1891 is an elegant book of over 100 pages, 8¼ x 10½ inches. The beautiful colored illustrations include Sunrise Amaranthus, Hydrangea and Potatoes. Instructions for planting, cultivating, etc. Full list of every thing that can de desired in the way of vegetable and flower seeds, plants, bulbs, etc. Also full particulars regarding the cash prizes of $1,000 and $200. The novelties have been tested and found worthy of cultivation. We advise our friends to secure a copy of James Vick, Seedsman, Rochester, N. Y.

